# KLF4 inhibited the senescence-associated secretory phenotype in ox-LDL-treated endothelial cells via PDGFRA/NAMPT/mitochondrial ROS

**DOI:** 10.18632/aging.205805

**Published:** 2024-05-08

**Authors:** Haoran Ding, Jing Tong, Hao Lin, Fan Ping, Tongqing Yao, Zi Ye, Jiapeng Chu, Deqiang Yuan, Kangwei Wang, Xuebo Liu, Fei Chen

**Affiliations:** 1 Department of Cardiology, Shanghai Tongji Hospital, School of Medicine, Tongji University, Shanghai 200092, China

**Keywords:** endothelial cells, SASP, KLF4, PDGFRA, NAMPT/mitochondrial ROS

## Abstract

Background: Inflammation is one of the significant consequences of ox-LDL-induced endothelial cell (EC) dysfunction. The senescence-associated secretory phenotype (SASP) is a critical source of inflammation factors. However, the molecular mechanism by which the SASP is regulated in ECs under ox-LDL conditions remains unknown.

Results: The level of SASP was increased in ox-LDL-treated ECs, which could be augmented by KLF4 knockdown whereas restored by KLF4 knock-in. Furthermore, we found that KLF4 directly promoted PDGFRA transcription and confirmed the central role of the NAPMT/mitochondrial ROS pathway in KLF4/PDGFRA-mediated inhibition of SASP. Animal experiments showed a higher SASP HFD-fed mice, compared with normal feed (ND)-fed mice, and the endothelium of EC-specific KLF4-/- mice exhibited a higher proportion of SA-β-gal-positive cells and lower PDGFRA/NAMPT expression.

Conclusions: Our results revealed that KLF4 inhibits the SASP of endothelial cells under ox-LDL conditions through the PDGFRA/NAMPT/mitochondrial ROS.

Methods: Ox-LDL-treated ECs and HFD-fed mice were used as endothelial senescence models *in vitro* and *in vivo*. SA-β-gal stain, detection of SAHF and the expression of inflammatory factors determined SASP and senescence of ECs. The direct interaction of KLF4 and PDGFRA promotor was analyzed by EMSA and fluorescent dual luciferase reporting analysis.

## INTRODUCTION

The vascular endothelium which is composed of an endothelial cell monolayer serves as the primary barrier against pro-atherosclerosis factors. Endothelial inflammation tends to induce secondary severe pathological changes and accelerate the progress of atherosclerosis [1]. Researchers have noted that senescent ECs may act as the most important inflammatory sources during atherosclerotic plaque formation, believed to further induce the release of interleukin 1 (IL-1), intracellular adhesion molecule 1 (ICAM-1), tumor necrosis factor (TNF-α), monocyte chemoattractant protein 1 (MCP-1) and other inflammatory factors [2], as an important characterization of the senescent-associated secretory phenotype (SASP). However, whether and how SASP is regulated in ECs in atherosclerosis remains unclear.

Krüppel-like factor 4 (KLF4) is a member of the SP/KLF family of transcription factors that contain a transactivation domain (TAD) and a repressor domain [3]. KLF4 is a housekeeper gene functioning in cycle progression, maintenance of a pluripotent stem cell state, cellular proliferation, and autophagy activity [4]. During atherosclerosis, KLF4 maintains normal vascular wall shear stress and vasopermeability and prevents the secretory phenotype switching from the contraction phenotype of smooth muscle cells and fibroblast inflammation [5, 6]. KLF4 also represses endothelial inflammation in vascular injury and neovascularization [7]. Nevertheless, whether and how KLF4 regulates ECs’ inflammatory factors release in SASP in atherosclerosis remains unknown.

In this study, we found that KLF4 was consistently down-regulated in both the vascular endothelium of HFD-fed mice and ox-LDL-treated ECs. And the gene encoding platelet-derived growth factor receptor α (PDGFRA), a receptor tyrosine kinase present in ECs and crucial for the development of chronic noninfective inflammation in several organs [8], was directly activated by KLF4. However, the function and molecular mechanism of the PDGFRA pathway in SASP of ECs remains unclear. Based on this, we explored whether PDGFRA pathway could function as a repressor of the SASP and verified PDGFRA pathway regulating nicotinamide phosphoribosyltransferase (NAMPT) and mitochondrial reactive oxygen (MitoROS) in endothelial cells. Altogether, we demonstrated that KLF4/PDGFRA/NAMPT/MitoROS pathway exerts a reliable inhibition of the SASP in endothelial cells in the ox-LDL-stimulated environment.

## RESULTS

### KLF4 inhibited the ox-LDL-induced EC SASP *in vivo* and *in vitro*

**Figure 1:**
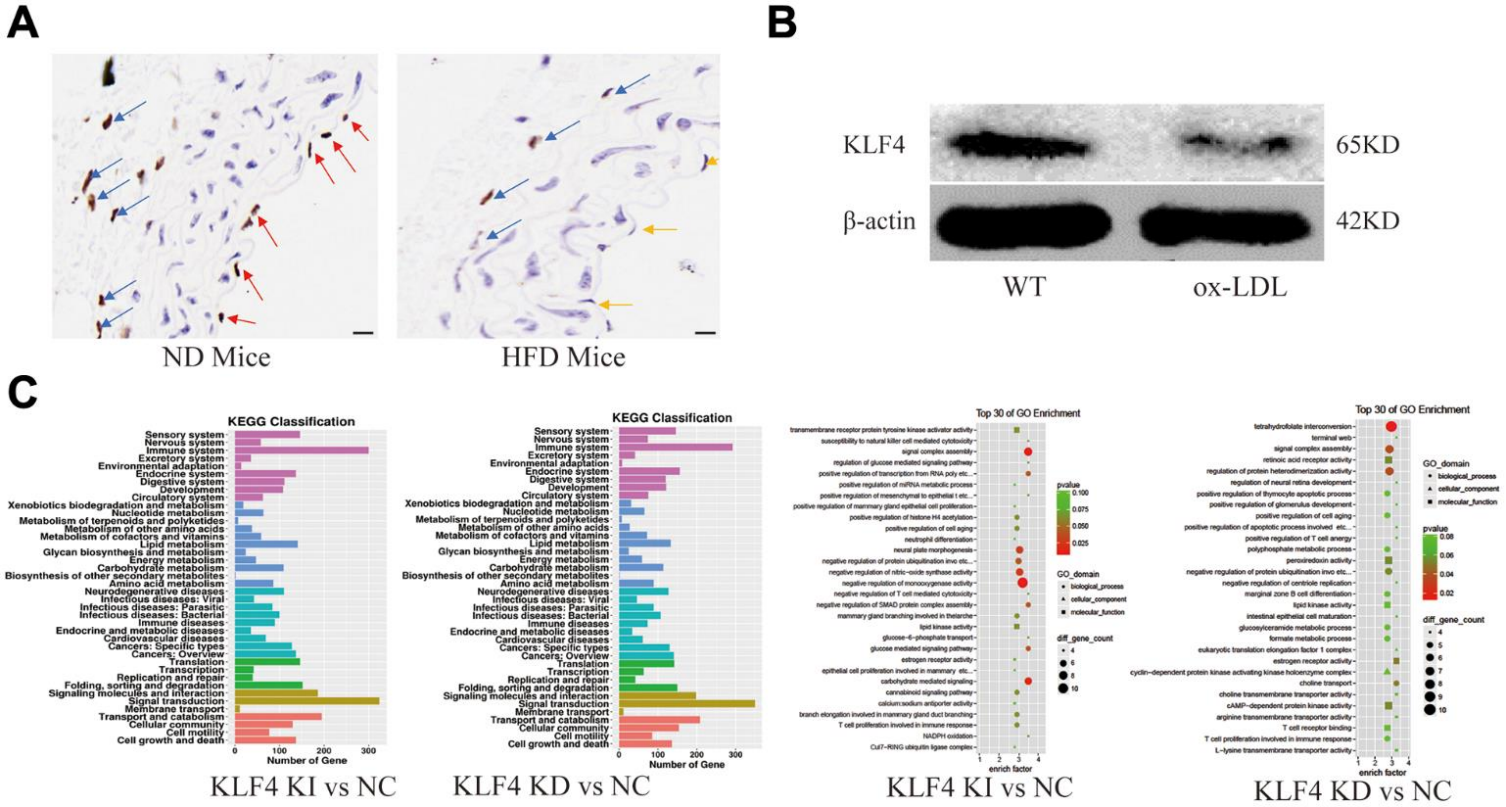
**Analysis of KLF4 protein expression and EC dysfunction.** (**A**) Immunohistochemical detection of vascular wall KLF4 expression. Scale bar, 20 μm. Representative images (n=5) are shown. Red arrow, KLF4 expression in the intima of mice fed a normal diet. Yellow arrow, intima lacking KLF4 expression in mice fed a high-fat diet. Blue arrow, KLF4 expression in adventitial fibroblasts. (**B**) Western blotting analysis of KLF4 protein expression in cultured HUVECs (100 μg/ml ox-LDL, 24 h) (n=5). (**C**) GO and KEGG analysis of the pathways after KLF4 knock in and knock out in HUVECs.

After feeding C57BL/6J mice a high-fat diet for 8 weeks, we used histopathology to observe the distribution of KLF4-positive cells. An obvious decrease in KLF4 expression in endothelial cells and adventitial fibroblasts was observed in high-fat diet-fed mice compared to normal diet-fed mice, especially in endothelial cells (Figure 1A). Interestingly, KLF4 expression was lacking in smooth muscle cells. The results were also verified using *in vitro* experiments. Western blotting results showed significantly downregulated KLF4 protein expression in ox-LDL-treated HUVECs (Figure 1B). To verify the possible role of KLF4 in endothelial cells, we analyzed the mRNA-seq in NC HUVECs, KLF4 knock-in HUVECs and KLF4 knockdown KUVECs. GO and KEGG analysis revealed that multiple pathways related to endothelial cells function and aging-related were significantly differentially regulated along with KLF4 expression changes (Figure 1C). These results indicated that KLF4 may play an important role in ox-LDL intervention in endothelial cells *in vivo* and *in vitro*.

**Figure 2:**
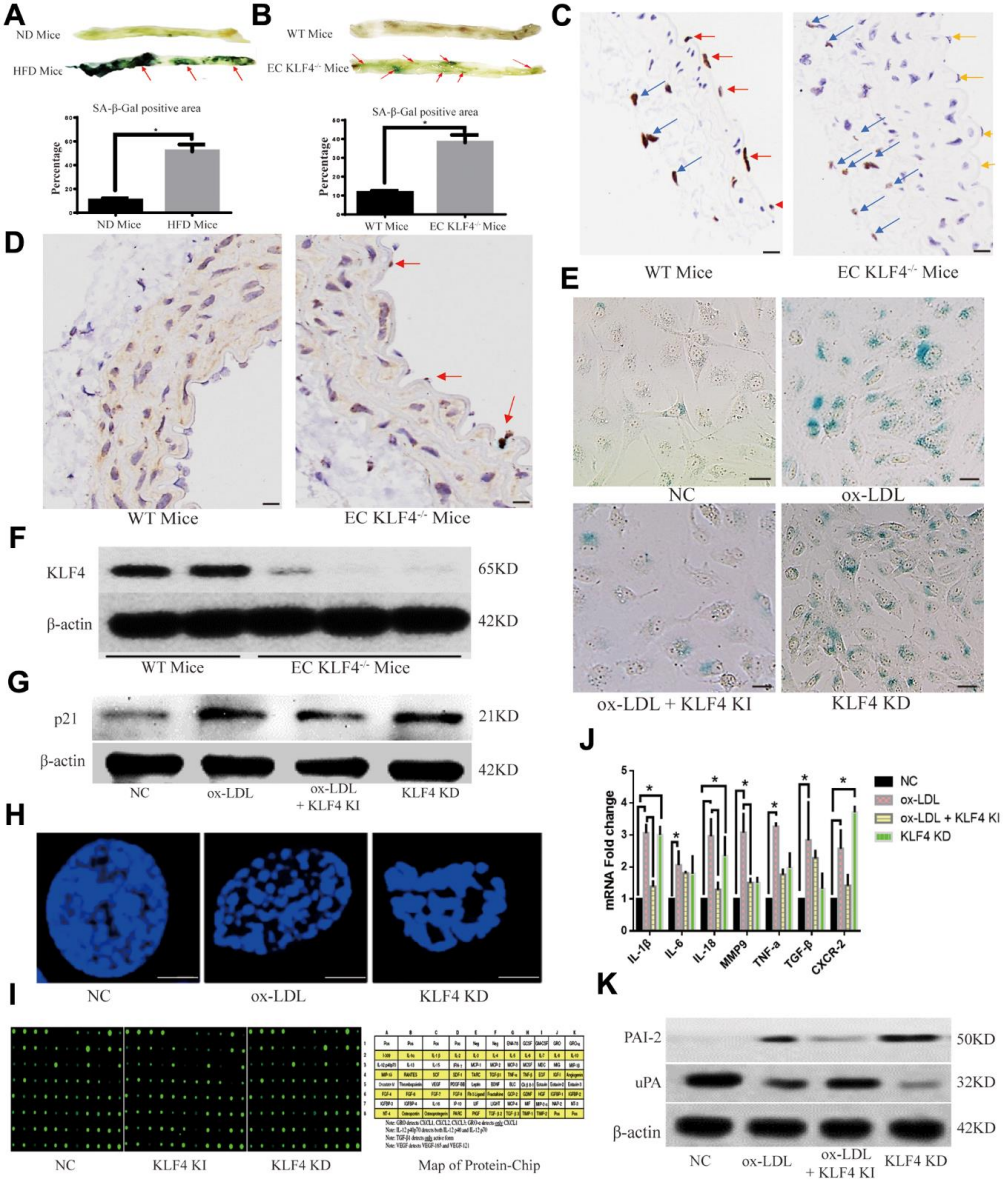
**KLF4 inhibits the EC SASP.** (**A**) Histochemical detection of the SA-β-gal-positive area in the vascular wall of mice fed a high-fat diet. Representative images (n=5) are shown. Red arrow, SA-β-gal-positive intima. (**B**) Immunohistochemical detection demonstrates the efficiency of specific conditional KLF4 knockout in mouse endothelial cells (EC KLF4^-/-^). Scale bar, 200 μm. Representative images (n=5) are shown. Red arrow, KLF4 expression in normal mouse intima. Yellow arrow, intima lacking KLF4 expression in mice fed a high-fat diet. Blue arrow, KLF4 expression in adventitial fibroblasts. (**C**) Histochemical detection of SA-β-gal-positive areas in the vascular wall of EC KLF4^-/-^ mice. Scale bar, 1 cm. Representative images (n=5) are shown. Red arrow, SA-β-gal-positive intima. (**D**) Immunohistochemical detection of p21 protein expression in the intima of EC KLF4^-/-^ mice. Scale bar, 200 μm. Representative images (n=5) are shown. Red arrow, p21-positive endothelial cells. (**E**) Histochemical detection of SA-β-gal-positive ECs in HUVECs. Scale bar, 50 μm. Representative images (n=5) are shown. Blue, SA-β-gal-positive ECs. (**F**) Western blotting analysis of KLF4 protein expression in the intima of EC KLF4^-/-^ mice. (**G**) Western blotting analysis of p21 protein expression in HUVECs after KLF4 expression is altered (n=5). (**H**) Immunofluorescence detection of typical SAHF formation in HUVECs (n=5). Scale bar, 5 μm. (**I**) Protein chip analysis in HUVECs (n=3). (**J**) qPCR analysis of the mRNA levels of cytokines in HUVECs (n=5). *P < 0.05. (**K**) Western blotting analysis of PAI-2 and uPA protein expression in HUVECs (n=5).

In high-fat diet-fed C57BL/6J mice, in addition to decreased KLF4 levels in the intima, the senescent intima area also exhibited increased SA-β-gal staining (Figure 2A). To investigate the role of KLF4 in mediating intima senescence in high-fat diet-fed mice, we designed specific conditional EC KLF4 knockout (EC KLF4^-/-^) mice, and the knockout efficiency was confirmed by histopathology (Figure 2B) and Western blotting in purified vascular ECs (Figure 2F). Next, we found that EC KLF4^-/-^ mice fed a normal diet exhibited a larger SA-β-gal-positive area (Figure 2C) and increased expression of the senescence protein p21 (Figure 2D) in the intima. To further verify the role of KLF4 in EC senescence, we designed Adv-KLF4 and shRNA-KLF4 to regulate KLF4 expression *in vitro*. Then, we found that transfection of ox-LDL-treated HUVECs with Adv-KLF4 led to a lower percentage of SA-β-gal-positive cells and lower p21 protein expression of p21, whereas KLF4 knockdown led to the opposite results (Figure 2E, 2G).

Cytokine release, which is also referred to as the SASP, is the most important role of senescent cells, and the specific chromatin rearrangement, called senescence-associated heterochromatin foci (SAHF), may represent its structural basis. Next, immunofluorescence results showed that KLF4 shRNA or ox-LDL treatment induced typical heterochromatin foci (Figure 2H). Protein chip and qPCR were used to observe cytokine release, and different cytokine protein production (Figure 2I and Table 1) and mRNA levels (Figure 2J) were observed in Adv-KLF4-transduced HUVECs and shRNA-KLF4-transfected HUVECs. In our experiments, we also observed that protein levels of the SASP biomarker type 2 plasminogen activation inhibitor (PAI-2) were decreased in ox-LDL-treated HUVECs transduced with Adv-KLF4 but increased in shRNA-KLF4-transfected HUVECs, whereas the opposite results were obtained for the SASP inhibition biomarker uridylyl phosphate adenosine (uPA) (Figure 2K). All of these *in vivo* and *in vitro* results indicated that KLF4 has an inhibitory effect on the SASP in ECs under ox-LDL-treatment conditions.

**Table 1:** Protein chip analysis of cytokines in cultured HUVECs (n=3).

**Inflammatory factor**	**KLF4 KI OD ratio**	**KLF4 KD OD ratio**
GRO-alpha	1.724*	0.499*
IL-1alpha	0.968	0.623*
IL-1beta	0.548*	1.925*
IL-6	2.276*	0.716
MCP-1	0.23*	0.938
RANTES	0.956	1.684*
PDGF-BB	1.829*	0.246*

*P < 0.05.

### PDGFRA plays an essential role in KLF4-mediated regulation of the SASP

**Figure 3:**
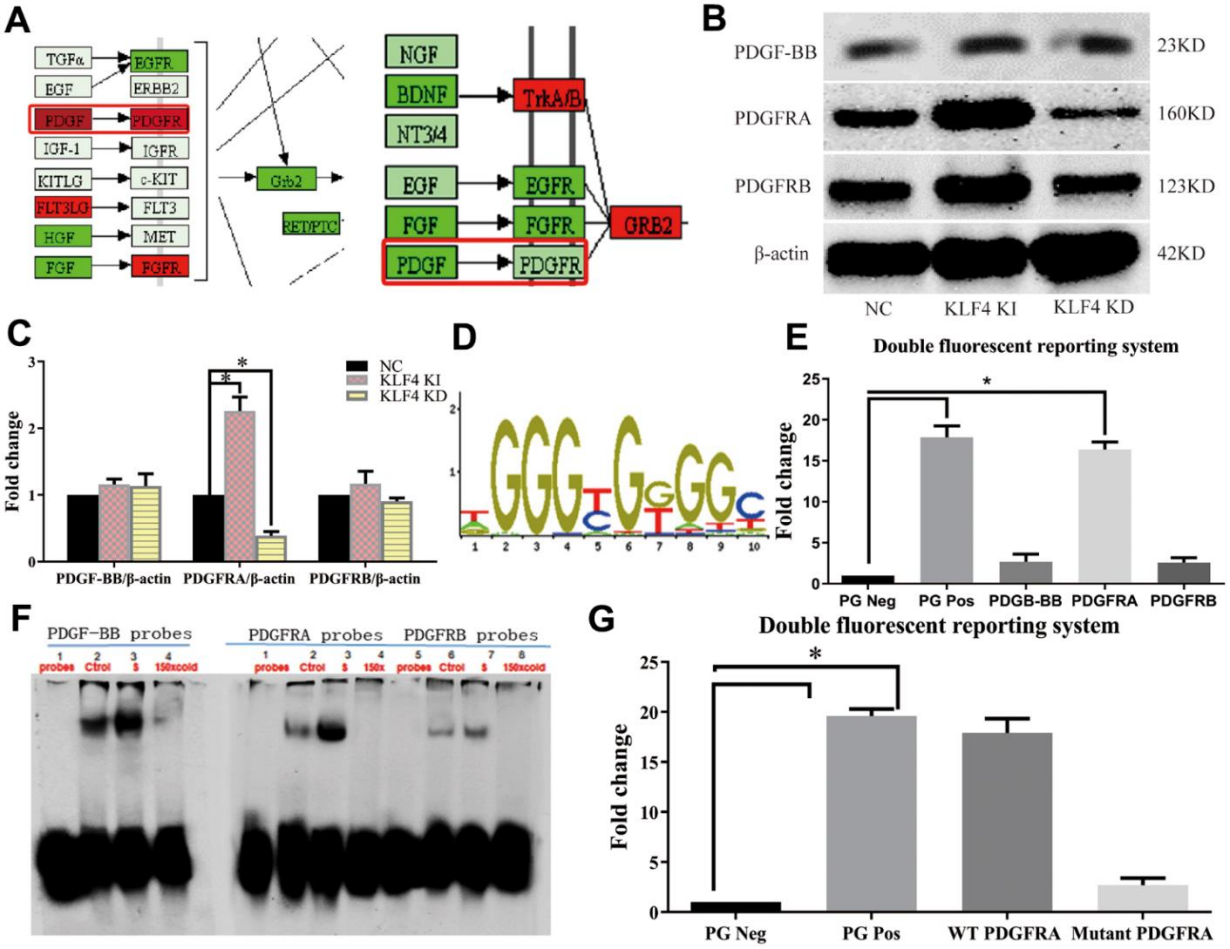
**KLF4 promotes PDGFRA transcription.** (**A**) PPI network analysis in cultured HUVECs. (**B**) Western blotting analysis of the PDGF pathway in HUVECs. (**C**) The amounts of PDGFRA, PDGF-BB and PDGFRB in (**B**) quantified using actin as control. (n=5). *P < 0.05. (**D**) Seed region for the KLF4 binding site. (**E**) Luciferase activity analysis of the PDGF pathway in HUVECs (n=5). *P < 0.05. (**F**) EMSA analysis of the PDGF pathway in HUVECs (n=3). *P < 0.05. (**G**) Luciferase activity of mutant PDGFRA in HUVECs (n=5). *P < 0.05.

**Figure 4:**
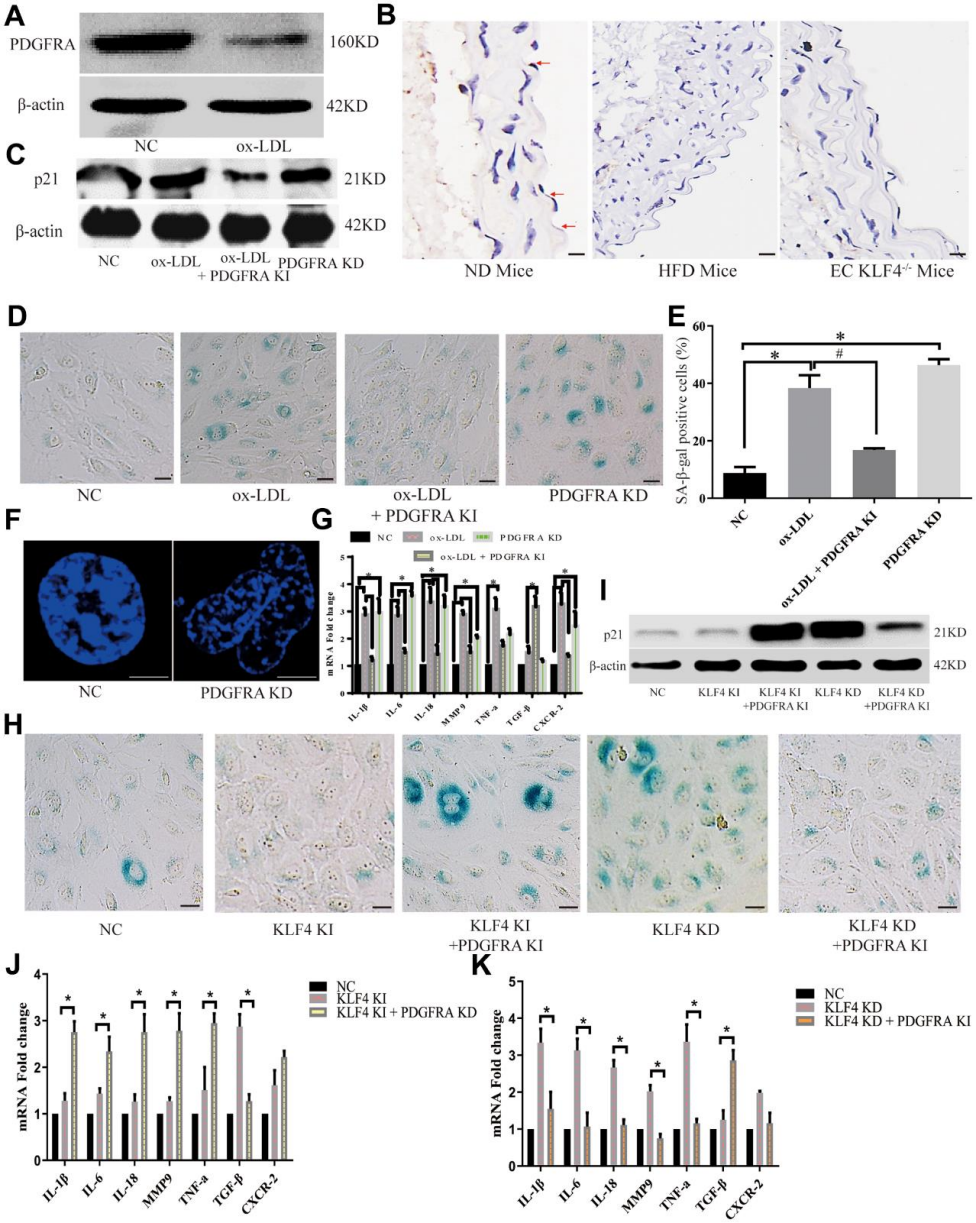
**KLF4 inhibited the HUVEC SASP through PDGFRA.** (**A**) Western blotting analysis of PDGFRA protein expression in ox-LDL-treated HUVECs (n=5). (**B**) Immunohistochemical detection of PDGFRA protein expression in the intima of ND-fed WT mice, HFD-fed WT mice and HFD-fed EC KLF4^-/-^ mice. Scale bar, 200 μm. Representative images (n=5) are shown. Red arrow, PDGFRA-positive endothelial cells. (**C**) Western blotting analysis of p21 protein expression in ox-LDL-treated HUVECs after altered PDGFRA expression (n=5). (**D**) Histochemical detection of SA-β-gal-positive ECs in ox-LDL-treated HUVECs after altered PDGFRA expression. Scale bar, 50 μm. Representative images (n=5) are shown. Blue, SA-β-gal-positive ECs. (**E**) SA-β-gal staining positive cells were counted and presented as percentage of total cells. (**F**) Immunofluorescence detection of typical SAHF formation in cultured HUVECs (n=5). Scale bar, 20 μm. (**G**) qPCR analysis of the mRNA levels of cytokines in ox-LDL-treated HUVECs after regulating PDGFRA (n=5). *P < 0.05. (**H**) Histochemical detection of SA-β-gal-positive ECs in KLF4-treated HUVECs after regulating PDGFRA. Scale bar, 50 μm. Representative images (n=5) are shown. Blue, SA-β-gal-positive ECs. (**I**) Western blotting analysis of p21 protein expression in KLF4-treated HUVECs after regulating PDGFRA (n=5). (**J**) qPCR analysis of the mRNA levels of cytokines in KLF4-knock-in HUVECs after PDGFRA knockdown (n=5). *P < 0.05. (**K**) qPCR analysis of cytokine mRNA levels in KLF4-knockdown HUVECs after PDGFRA knock-in (n=5). *P < 0.05.

In our experiments, we analyzed the protein-protein interaction (PPI) network in NC HUVECs, KLF4 knock-in HUVECs and KLF4 knockdown KUVECs. The PDGF/PDGFR pathway was increased in KLF4 knock-in HUVECs, whereas KLF4 shRNA-transfected HUVECs exhibited the opposite result (Figure 3A). To verify communication between KLF4 and the PDGF/PDGFR pathway, we observed the expression of proteins in the PDGF/PDGFR pathway and found that only PDGFRA protein expression was increased in Adv-KLF4-transduced HUVECs. Moreover, PDGFRA protein expression was decreased in shRNA-KLF4-transfected HUVECs (Figure 3B). To further verify whether KLF4 directly promoted PDGFRA transcription, we searched the seed region (Figure 3C) and designed pGL4 probes and biotin probes for *in vitro* experiments. We found that transfection of the pGL4-KLF4 vector with PDGFRA, but not platelet-derived growth factor-BB (PDGF-BB) or platelet-derived growth factor receptor β (PDGFRB), into HUVECs effectively induced luciferase activity (Figure 3D). The EMSA results also showed that KLF4 tightly bound to biotin-labeled PDGFRA but not PDGF-BB or PDGFRB (Figure 3E). Next, we designed a PDGFRA mutant in the KLF4 binding seed region with a poly-A sequence and then transfected it with the pGL4-KLF4 vector; no luciferase activity was observed (Figure 3F). These findings indicated that KLF4 promotes PDGFRA transcription. We wanted to assess whether communication occurs between KLF4 and PDGFRA in the KLF4-regulated SASP. To confirm this hypothesis, we observed PDGFRA protein expression in ECs and found that PDGFRA protein expression decreased in ox-LDL-treated HUVECs (Figure 4A) and high-fat diet-fed mice (Figure 4B). Histopathology showed that PDGFRA protein expression was also reduced in EC KLF4^-/-^ intima in mice fed a normal diet (Figure 4B). Next, we used Adv-PDGFRA and shRNA-PDGFRA to upregulate and downregulate PDGFRA expression, respectively, in non-treated and ox-LDL-treated HUVECs. PDGFRA overexpression in ox-LDL-treated HUVECs led to an apparent decrease in p21 protein expression (Figure 4C), a decrease in the percentage of SA-β-gal-positive HUVECs (Figure 4D), and a reduced level of cytokine secretion (Figure 4F). Reduced PDGFRA expression had the opposite effect (Figure 4C–4F). Then, we downregulated PDGFRA expression in KLF4 knock-in HUVECs by transfecting PDGFRA shRNA and restored the HUVEC SASP. In the context of reduced PDGFRA expression, the percentage of SA-β-gal-positive HUVECs (Figure 4G), p21 protein expression (Figure 4H), typical SAHF structure formation (Figure 4I) and cytokine release (Figure 4J) exhibited sharp increases in KLF4 knock-in HUVECs. Then, we overexpressed PDGFRA in KLF4 knockdown HUVECs by transfecting Adv-PDGFRA. Here, the increase in the SASP in KLF4-knockdown HUVECs was attenuated by the overexpression of PDGFRA (Figure 4G–4I, 4K). These results confirmed that the regulatory role of KLF4 on the SASP is at least partly mediated by PDGFRA.

### The NAMPT/mitochondrial ROS pathway participates in KLF4/PDGFRA-mediated inhibition of the SASP in ox-LDL-treated HUVECs

**Figure 5:**
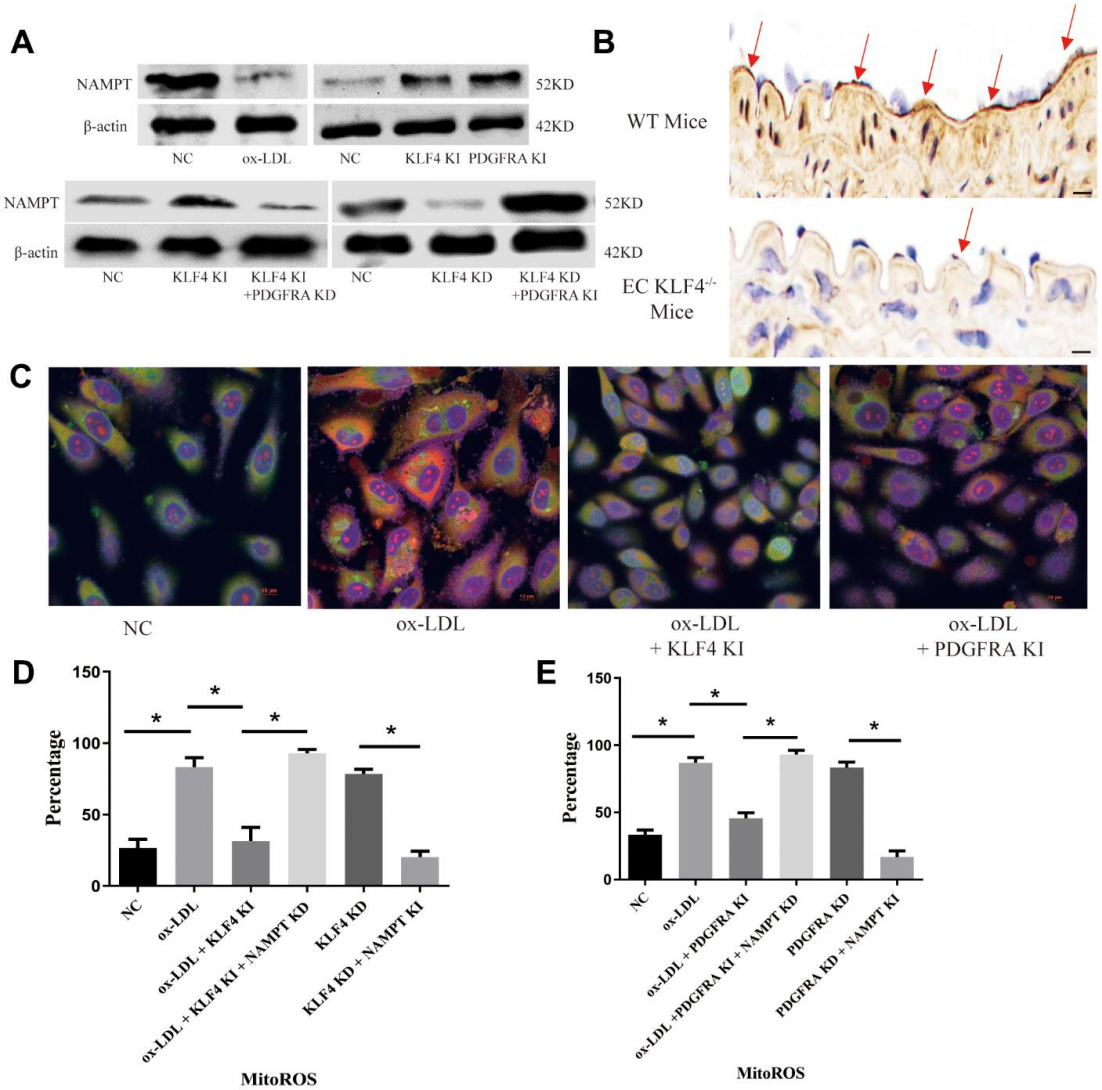
**KLF4/PDGFRA regulated NAMPT/MitoROS expression.** (**A**) Western blotting analysis of NAMPT protein expression in cultured HUVECs (n=5). (**B**) Immunohistochemical detection of NAMPT protein expression in the intima of high-fat diet-fed mice or EC KLF4^-/-^ mice. Scale bar, 200 μm. Representative images (n=5) are shown. Red arrow, NAMPT-positive endothelial cells. (**C**) Immunofluorescence detection of MitoROS in cultured HUVECs. Scale bar, 20 μm. Representative images (n=5) are shown. Red, ROS. Green, Mitochondria. Yellow, MitoROS. (**D**) Flow cytometry analysis for MitoROS quantification in KLF4-treated HUVECs after altering NAMPT expression (n=5). *P < 0.05. (**E**) Flow cytometry analysis of MitoROS quantification in PDGFRA-treated HUVECs after altering NAMPT expression (n=5). *P < 0.05.

NAMPT-mediated mitochondrial ROS (MitoROS) production is a regulator of chronic inflammation regulator as documented in numerous studies. In our experiments, we observed NAMPT protein expression changes under different conditions. We found that NAMPT protein expression decreased in ox-LDL-treated HUVECs (Figure 5A) and high-fat diet-fed mice (Figure 5B), whereas KLF4 or PDGFRA overexpression increased NAMPT protein expression (Figure 5A). Moreover, histopathological assessment revealed that intima NAMPT protein expression was decreased in KLF4^-/-^ EC mice (Figure 5B). Then, we downregulated PDGFRA in KLF4 knock-in HUVECs by transfecting PDGFRA shRNA and assessed NAMPT protein expression. Here, reduced PDGFRA expression resulted in reduced NAMPT protein expression (Figure 5A). Next, we overexpressed PDGFRA in KLF4 knockdown HUVECs by adding Adv-PDGFRA and obtained the opposite results (Figure 5A). Then, we used MitoSOX to detect MitoROS and found that ox-LDL-treated HUVECs had higher MitoROS levels, whereas Adv-KLF4- or Adv-PDGFRA-transduced HUVECs had lower MitoROS levels (Figure 5C). In addition, we separately downregulated NAMPT in KLF4 knock-in or PDGFRA knock-in HUVECs and overexpressed NAMPT in KLF4 knockdown or PDGFRA knockdown HUVECs and measured MitoROS levels. We observed lower MitoROS levels along with higher NAMPT expression in KLF4 knockdown or PDGFRA knockdown HUVECs, whereas lower NAMPT expression and higher MitoROS levels were noted in KLF4 knock-in or PDGFRA knock-in HUVECs (Figure 5D, 5E). These results confirmed that KLF4/PDGFRA regulated MitoROS production partially through NAMPT.

**Figure 6:**
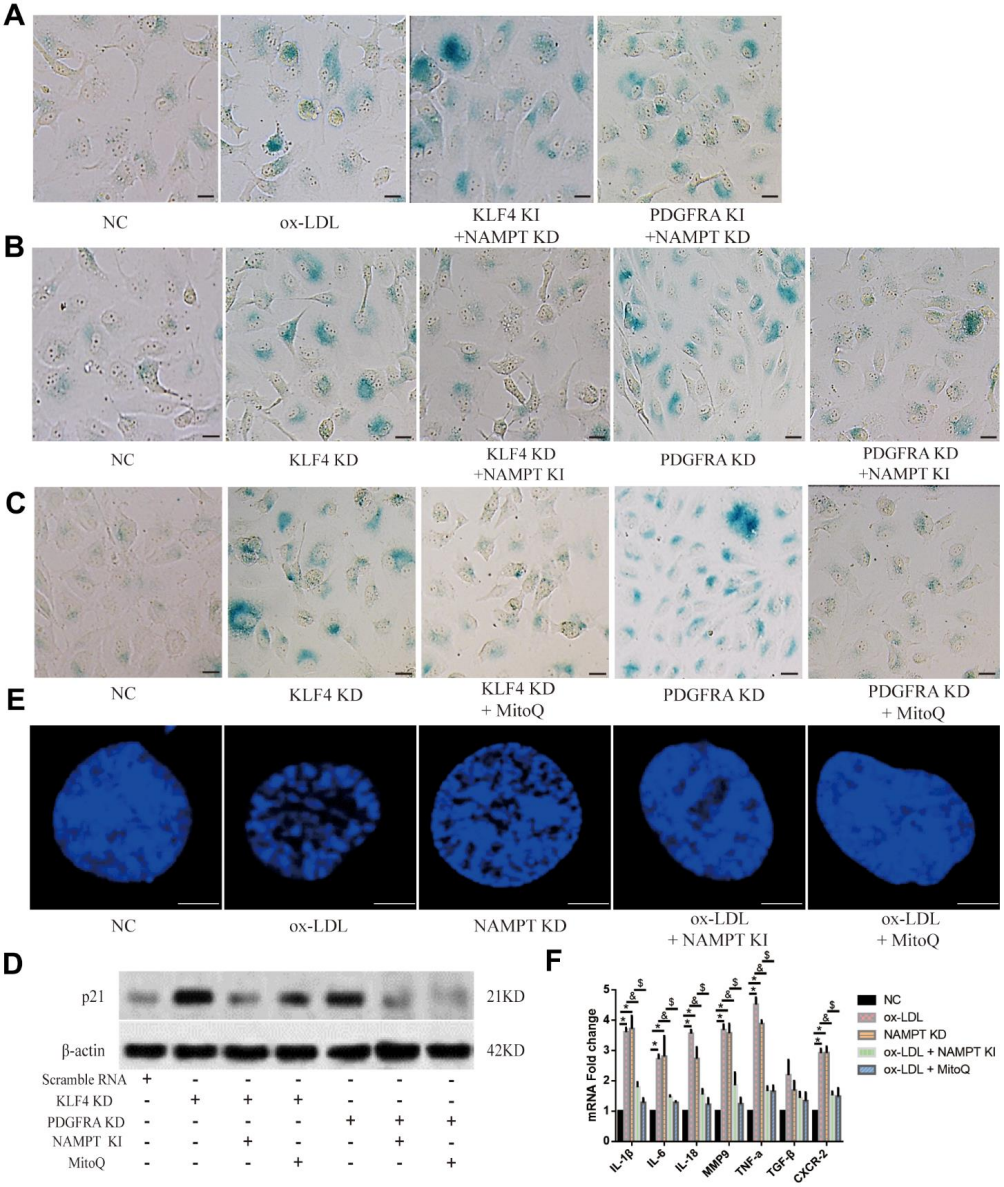
**NAMPT/MitoROS regulated the SASP in HUVECs.** (**A**) Histochemical detection of SA-β-gal-positive ECs in NAMPT knockdown HUVECs after knock-in of KLF4 or PDGFRA. Scale bar, 50 μm. Representative images (n=5) are shown. Blue, SA-β-gal-positive ECs. (**B**) Histochemical detection of SA-β-gal-positive ECs in NAMPT knock-in HUVECs after KLF4 or PDGFRA knockdown. Scale bar, 50 μm. Representative images (n=5) are shown. Blue, SA-β-gal-positive ECs. (**C**) Histochemical detection of SA-β-gal-positive ECs in MitoQ-treated HUVECs after KLF4 or PDGFRA knockdown. Scale bar, 50 μm. Representative images (n=5) are shown. Blue, SA-β-gal-positive ECs. (**D**) Western blotting analysis of p21 protein expression in NAMPT knock-in- or MitoQ-treated HUVECs (n=5). (**E**) Immunofluorescence detection of typical SAHF formation in cultured HUVECs after altering NAMPT expression or treatment with MitoQ (n=5). Scale bar, 20 μm. (**F**) qPCR analysis of cytokine mRNA levels in cultured HUVECs after altering NAMPT expression or treatment with MitoQ (n=5). *P < 0.05.

To determine the potential role of NAMPT/MitoROS in the HUVEC SASP, we performed SA-β-gal staining and found that NAMPT knockdown increased the SA-β-gal-positive staining percentage in Adv-KLF4-treated or Adv-PDGFRA-treated HUVECs (Figure 6A). In addition, NAMPT overexpression yielded the opposite result in KLF4 shRNA-treated HUVECs or PDGFRA shRNA-treated HUVECs (Figure 6B). Next, we used Mitoquinone Q (MitoQ, 1 μM) treatment to scavenge MitoROS and assessed SA-β-gal-positive HUVECs. We found that MitoQ treatment decreased the SA-β-gal-positive staining percentage in KLF4 shRNA-treated HUVECs and PDGFRA shRNA-treated HUVECs (Figure 6C). Then, we assessed p21 protein expression and found that NAMPT knock-in or MitoQ treatment reduced p21 protein expression in KLF4 shRNA-transfected HUVECs or PDGFRA shRNA-transfected HUVECs (Figure 6D). Next, the immunofluorescence results showed that NAMPT knock-in or MitoQ treatment prevented typical SAHF formation in ox-LDL-treated HUVECs, whereas knockdown of NAMPT mediated typical SAHF formation (Figure 6E). Then, we assessed cytokine mRNA levels. NAMPT knock-in or MitoQ treatment decreased cytokine mRNA levels in ox-LDL-treated HUVECs, whereas NAMPT knockdown increased cytokine mRNA levels (Figure 6F). These results demonstrated that NAMPT/MitoROS are involved in KLF4/PDGFRA-mediated SASP inhibition in ox-LDL-treated HUVECs.

## DISCUSSION

Atherosclerosis remains a leading cause of morbidity and mortality worldwide, and determining the underlying pathophysiological mechanisms is crucial for the development of new therapeutic strategies [9]. In recent years, the effect of senescence, especially the SASP, in 3 types of intravascular cells on atherosclerosis has become a research hotspot [10]. Research documents have shown that senescent SMCs and ECs together with the SASP are increased in the fibrous cap of atherosclerotic plaques [11], and secreted cytokines contribute to collagen degradation, lipid uptake, the accumulation of foam cells, plaque rupture and thrombosis [12]. Of note, the endothelial cell SASP is proposed to be the initial event in the development of atherogenesis [13], but its mechanism has not been completely characterized. In this study, we found a relationship between KLF4 and the SASP in ox-LDL-induced endothelial cells and high-fat diet-fed mouse intima and investigated the possible role of PDGFRA through which KLF4 exerts a regulatory function on the SASP. Our experimental results suggested that KLF4 directly promoted PDGFRA transcription and then prevented the SASP in ox-LDL-treated ECs. Intriguingly, KLF4/PDGFRA overexpression led to increased expression of NAMPT and decreased expression of MitoROS. NAMPT overexpression or elimination of MitoROS attenuates the SASP in ox-LDL-treated, KLF4 knockdown or PDGFRA knockdown HUVECs, and the opposite effect was obtained with NAMPT knockdown. These results indicated that KLF4/PDGFRA-mediated SASP suppression partially involves the NAMPT/MitoROS pathway in ox-LDL-treated HUVECs.

Of note, KLF4 may induce positive or negative effects of senescence during atherosclerosis because it exhibits dual regulation of protein expression through direct DNA binding [3, 14]. Given that KLF4 has been reported to directly or indirectly promote p21 transcription [15, 16], some research suggests that KLF4 may act as a cell cycle repressor and senescence inducer [17]. In endothelial cells, KLF4 has also been demonstrated to induce pathologic senescence and the SASP through a p21-dependent manner in high glucose-treated endothelial cells [18]. However, it should be noted that KLF4 typically functions as an inducer of cellular pluripotency [19], and most studies have confirmed that KLF4 overexpression is very important to maintain a young cellular phenotype and suppress replicative senescence and physiological or pathological aging [4, 7, 20, 21]. Additionally, some papers reported that p21 could also alter KLF4 expression in an acetylation-dependent manner [5] or induce KLF4 translocation into the cytoplasm [22]. Therefore, we hypothesized that KLF4-regulated senescence may occur in a context-dependent manner and depend on the cell type or the stress microenvironment. In our previous study, we revealed that KLF4 induced autophagy in advanced glycation end products (AGE)-treated or ox-LDL-treated HUVECs [23], and autophagy has been reported to restrict senescence in multiple cell types [24]. Lipid metabolism dysfunction is the key risk factor for atherogenesis. Thus, in this study, we assessed the effect of KLF4 in an ox-LDL-treated HUVEC SASP model and a high-fat diet-induced intima senescence model and found that ox-LDL or a high-fat diet significantly decreased KLF4 protein expression. Interestingly, we found that KLF4 inhibits p21 expression in the present study. This finding is inconsistent with previous observations in high glucose-treated HUVECs [18] but consistent with observations in stem cells and cancer cells [25]. This difference may be explained by the notion that high glucose levels occasionally induce cell proliferation and replicative senescence. In the present study, the effects of ox-LDL levels did not mimic by the inflammatory control induced by LPS. Thus, endothelial cell uptake and lipid metabolism are prerequisites for ox-LDL-induced endothelial SASP. Our novel findings may provide new molecular insights into the regulation of endothelial SASP in atherosclerosis.

In this study, we also explored the downstream signaling targets of KLF4 in an ox-LDL-induced SASP model and assessed the role of its downstream target PDGFRA. Although direct evidence to inhibit senescence is lacking, studies have demonstrated that the receptor tyrosine protein kinase PDGFRA is a basic protein that stimulates multiple cell types to enter the proliferative cycle and growth, inducing cell differentiation and development [26–28]. In endothelial cells, PDGFRA overexpression also resulted in the reversion of phenotypes and induced proliferation and angiogenesis [29]. These data suggested that PDGFRA may have the ability to inhibit senescence. Recently, research revealed that PDGFRA inhibitor-treated cells showed increased senescence marker levels [30] and p53 protein expression [31]. In the present study, we explored the pathophysiological action of PDGFRA in an ox-LDL-induced HUVEC senescence model and found that PDGFRA overexpression significantly reduced the number of SA-β-gal-positive cells or typical SAHF cell formation. Thus, these data provide direct evidence that PDGFRA may represent a key anti-senescence factor in an ox-LDL environment. As discussed in the Introduction, the major pathological effects of senescent cells may be attributed to the SASP [2]. Then, we assessed cytokine secretion after PDGFRA treatment in ox-LDL-treated HUVECs and found that PDGFRA inhibited proinflammatory cytokine release in ox-LDL-treated HUVECs. These data are consistent with its anti-inflammatory role in cancer cells, immune cells, fibroblasts and cardiac stromal cells [8, 32, 33]. Although most studies support that PDGFRA and PDGF exhibit classical receptor-ligand binding interactions to perform their pro-proliferation role in cancer cells [34], it should be noted that PDGFRA also exerts its role in a ligand-independent manner through various processes, such as autophosphorylation [35]. In our present study, protein chip experiments showed that PDGF-BB secretion was altered in KLF4-regulated HUVECs, whereas bioinformatics analysis and molecular biology experiments indicated that KLF4 directly targeted the increase in PDGFRA protein expression in ox-LDL-treated HUVECs. However, the process was not dependent on PDGF-BB binding. Furthermore, increased PDGFRA expression may be due to the direct transcriptional promotion of PDGFRA by KLF4. This regulatory network would be consistent with the notion that “PDGF-independent PDGFR activation may play more key role in much more story” [36] and the phenomenon wherein “PDGFRA expression increased together with KLF4 expression in stem cells” [37].

An increasing number of studies have found that reactive oxygen species (ROS) may be the major inducer of vascular senescence, and the NAD^+^/mitochondrial ROS (MitoROS) pathway seems to be the most important pathway for ROS production [38]. NAMPT is the rate-limiting enzyme in the NAD^+^/MitoROS metabolic pathway [39], and the “NAD world” comprising “NAMPT/NAD^+^/MitoROS/Sirt1” may represent an intact loop that modulates mammalian aging processes [40]. Previous studies showed that NAMPT-deficient cells exhibited a higher percentage of SA-β-gal-positive cells, increased inflammatory factor levels and more senescence-associated gene expression [41]. Based on the above findings, we performed NAMPT/MitoROS functional experiments in ox-LDL-treated HUVECs. The current study showed that ox-LDL-induced HUVEC senescence also relied on NAMPT/MitoROS scavenging, and NAMPT overexpression or MitoROS removal impeded SAHF formation and cytokine secretion in ox-LDL-treated HUVECs. Although direct evidence of NAMPT in KLF4-inhibited senescence is lacking, previous studies verified that KLF4 plays a key role in mitochondrial biogenesis and MitoROS production [42]. KLF4 activates PGC-1a to remove MitoROS and initiates mitophagy to maintain mitochondrial homeostasis [6]. KLF4-deficient cells exhibited abnormal mitochondrial accumulation, superfluous MitoROS production, and abundant inflammatory factor secretion [42, 43]. Therefore, we hypothesized that KLF4 inhibited HUVEC senescence through the NAMPT/MitoROS pathway. Our experimental results showed that scavenging MitoROS reversed the young phenotype in KLF4 knockdown HUVECs, whereas NAMPT knockdown accelerated HUVEC senescence and the SASP phenotype in KLF4 knock-in HUVECs. Interestingly, a previous study revealed that extracellular NAMPT also participated in PDGF pathway-induced pulmonary smooth muscle cell proliferation [44]. Considering the relationship between PDGFRA and SASP, we also altered NAMPT expression in PDGFRA knock-in or PDGFRA knockdown HUVECs, and the results showed that intracellular knockdown of NAMPT mediated the SASP phenotype in PDGFRA knock-in HUVECs. These results suggest that KLF4/PDGFRA signaling plays a key role in regulating SASP via NAMPT-MitoROS metabolism and may function in a ligand-independent manner to activate PDGFA in ox-LDL-treated ECs. Finally, it should be noted that MitoROS was the most powerful inducer of p21 protein expression [45], which may partially explain the different expression patterns of KLF4 and p21 protein in our experiment.

In conclusion, KLF4 exerts a negative role in regulating the SASP process in endothelial cells *in vitro* and *in vivo*, and this function is carried out via PDGFRA. The NAMPT/MitoROS pathway partially participates in the KLF4/PDGFRA-regulated SASP. Given that the SASP is an increasingly important process in inducing atherosclerosis formation and development, the results from the current study provide new molecular insights into the SASP in endothelial cells in the context of atherosclerosis.

## MATERIALS AND METHODS

### Animals

All animal testing procedures were performed in accordance with the Guide for the Care and Use of Laboratory Animals published by the US National Institutes of Health (NIH revised 2011) and approved by the Institutional Animal Care and Use Committee of Tongji University. Eight-week-old C57BL/6J male mice were purchased from the Experimental Animal Centre, Chinese Academy of Sciences (Shanghai, China). Specific conditional EC KLF4 knockout mice were established by Saiye Corp. (Suzhou, Jiangsu, China). Briefly, we crossbred Tek-Cre: KLF4 and Floxp/Floxp mice. Genotyping was performed at 2 weeks after birth. The Tek-Cre: KLF4/Floxp mice have specific KLF4 deletion ECs (called KLF4^-/-^ ECs). After being fed a normal diet or a high-fat diet for 8 weeks, the mice were anesthetized using isoflurane and euthanized using cervical dislocation.

### Reagents

Adv-vectors (KLF4, PDGFRA and NAMPT) were used to overexpress target genes, and sh-RNAs (KLF4, PDGFRA and NAMPT) were used to knock down target genes. All were purchased from Viogene Bio. (Jinan, Shandong, China). The KLF4 luciferase reporter plasmid (pGL4-KLF4) was purchased from Yeasen Bio. (Shanghai, China). A fluorescent dual luciferase reporting system and murine leukemia virus reverse transcriptase were obtained from Promega Co. (Mannheim, Germany). The SA-β-gal-kit was obtained from Beyotime (Haimen, China). The MitoSOX Mitochondrial Superoxide Indicator was obtained from Yeasen (Shanghai, China). The anti-p21 antibody was obtained from HUABIO (Hangzhou, China). The anti-PDGF-BB, anti-PDGFRA, anti-PDGFRB, anti-KLF4, anti-NAMPT, anti-PAI-2, anti-uPA and anti-β-actin antibodies and secondary antibodies were obtained from Cell Signaling Technology (Danvers, MA, USA). Other reagents were purchased from Sigma (St. Louis, MO, USA).

### Cell culture

The human umbilical vein endothelial cell (HUVEC) line and endothelial cell media (ECM) were obtained from ScienceCell (Carlsbad, CA, USA). HUVECs were grown in ECM (glucose: 5.5 mM). After starvation by FBS deprivation for 12 h, HUVECs were exposed to ox-LDL (100 μg/ml) for 24 h to induce senescence.

### Real-time reverse transcription PCR (RT-PCR)

Total RNA was converted to cDNA using the murine leukemia virus reverse transcriptase system. RT-PCR was performed using an ABI 7500 system and SYBR Premix chemistry (Takara, Shiga, Japan) following the manufacturer’s instructions. β-Actin was chosen as an endogenous expression standard. The cycle number represents the relative quantity of the specific template when the fluorescence of the amplified gene product first reached a preset threshold (Ct). Gene expression levels were calculated using the double delta Ct method. All amplifications were performed independently three different times. The following primers were used (Sangon Bio., Shanghai, China): IL-1β: Forward: 5’-ATGATGGCTTATTACAGTGGCAA-3’, Reverse: 5’-GTCGGAGATTCGTAGCTGGA-3’; IL-6: Forward: 5’-ACTCACCTCTTCAGAACGAATTG-3’, Reverse: 5’-CCATCTTTGGAAGGTTCAGGTTG-3’; IL-18: Forward: 5’-TTGGCCCAGGAACAATGGCTGC-3’, Reverse: 5’-TGCGGTTGTACAGTGAAGTCGG-3’; MMP9: Forward: 5’-CAGTACCGAGAGAAAGCCTATT-3’, Reverse: 5’-CAGGATGTCATAGGTCACGTAG-3’; TNF-a: Forward: 5’-TGCACTTTGGAGTGATCGGC-3’, Reverse: 5’-AGCTTGAGGGTTTGCTACAAC-3’; TGF-β: Forward: 5’-CCGTCTCCTACCAGACCAAGG-3’, Reverse: 5’-CCGTCTCCTACCAGACCAAGG-3’; CXCR-2: Forward: 5’-AAGGTGAATGGCTGGATTTTTG-3’, Reverse: 5’-CCCAGATGCTGAGACATATGAA-3’.

### Protein extraction and Western blot

Cultured HUVECs or mouse aortic walls were lysed in RIPA buffer (EpiZyme, Shanghai, China) and sonicated briefly before protein extraction. Proteins were quantified using a bicinchoninic acid (BCA) protein assay kit (EpiZyme, Shanghai, China). Proteins (40 μg) were separated on SDS-PAGE gels and transferred onto 0.45-μm PVDF membranes (Millipore, Burlington, MA, USA), which were then incubated with primary antibodies at 4° C overnight after blocking with 5% fat-free milk in TBST buffer. On the following day after incubation with HRP-conjugated secondary antibodies for 1 h at room temperature, the membranes were visualized using enhanced ECL reagent (Share-Bio, Shanghai, China). The relative intensities of the protein bands were analyzed using ImageJ software.

### SA-β-gal analysis

SA-β-gal analysis was performed according the SA-β-gal kit manufacturer’s instructions. Briefly, after fixation for 15 min, fresh aortic walls or cells were incubated with 5-bromo-4-chloro-3-inolyl-b-D-galactoside (β-gal) substrate for 24 h at 37° C and then imaged.

### SAHF detection

After fixation with 90% ice-cold methanol for 2 min, the cells were stained with 0.1 μg/mL anti-fade DAPI for 2 min and then analyzed using a laser confocal microscope.

### EMSA

Nucleoproteins were obtained from cultured HUVECs. After incubation with specific probes with biotin, the samples were resolved by TBE-PAGE and then visualized using a streptavidin-HRP conjugate.

The following probes (1000 fmol) were used:

PDGF-BB, bio5’-gcttgttaccacacccagctccag-3’, 3’-cgaacaatggtgtgggtcgaggtc-5’-bio;

PDGFRA, bio5’-caggcgcaaccaggcccaggtggc-3’, 3’-gtccgcgttggtccgggtccaccg-5’-bio;

PDGFRB, bio5’-gttgaggctgggtgcggtggctcaag-3’, 3’-caactccgacccacgccaccgagttc-5’-bio.

### Fluorescent dual luciferase reporting analysis

For luciferase analysis, the pGL4-KLF4 vector and PDGF-BB, PDGFRA and PDGFRB vectors were cotransfected, and cells were incubated for 48 h. The samples were collected and subjected to 3 freeze-thaw cycles. The results were obtained using a Lumat LB9507 luminometer (EG and GBerthold). Transfection efficiency was normalized to Renilla luciferase.

### mRNA-seq array

An mRNA-seq array of total RNA obtained from HUVECs was performed by Riobio Tech, Inc. (Guangzhou, China).

### Inflammatory factor spectrum array

An inflammatory factor spectrum array from total proteins obtained from lysing HUVECs was performed by Riobio Tech, Inc. (Guangzhou, China).

### Histopathology

For histological analysis, freshly dissected aortas were fixed in 10% (v/v) formalin for 24 h, embedded in paraffin, sliced into 4-mm thick sections, stained with β-gal and specific primary antibodies and observed using a ZEISS AXIO scanning microscope.

### MitoROS detection

MitoROS analysis was performed according to the MitoSOX kit manufacturer’s instructions. Briefly, freshly cultured HUVECs were first stained with a mitochondria staining kit, and then the MitoSOX stain was added. Samples were incubated at 37° C for 15 min. The samples were analyzed using a laser confocal microscope or flow cytometry.

### Statistical analysis

Statistical analysis was performed using SPSS 22.0 software. All data are presented as the mean ± SEM and were compared using one-way ANOVA followed by Tukey’s HSD test. A value of *P* < 0.05 was considered to indicate statistical significance.

## References

[1] Meng B, Li Y, Ding Y, Xu X, Wang L, Guo B, Zhu B, Zhang J, Xiang L, Dong J, Liu M, Xiang L, Xiang G. Myeloid-derived growth factor inhibits inflammation and alleviates endothelial injury and atherosclerosis in mice. Sci Adv. 2021; 7:eabe6903. 10.1126/sciadv.abe6903 34020949 PMC8139583

[2] Ma S, Fan L, Cao F. Combating cellular senescence by sirtuins: Implications for atherosclerosis. Biochim Biophys Acta Mol Basis Dis. 2019; 1865:1822–30. 10.1016/j.bbadis.2018.06.011 29944946

[3] Yu T, Chen X, Zhang W, Liu J, Avdiushko R, Napier DL, Liu AX, Neltner JM, Wang C, Cohen D, Liu C. KLF4 regulates adult lung tumor-initiating cells and represses K-Ras-mediated lung cancer. Cell Death Differ. 2016; 23:207–15. 10.1038/cdd.2015.85 26113043 PMC4716302

[4] Panatta E, Lena AM, Mancini M, Affinati M, Smirnov A, Annicchiarico-Petruzzelli M, Piro MC, Campione E, Bianchi L, Mazzanti C, Melino G, Candi E. Kruppel-like factor 4 regulates keratinocyte senescence. Biochem Biophys Res Commun. 2018; 499:389–95. 10.1016/j.bbrc.2018.03.172 29580988

[5] Salmon M, Gomez D, Greene E, Shankman L, Owens GK. Cooperative binding of KLF4, pELK-1, and HDAC2 to a G/C repressor element in the SM22α promoter mediates transcriptional silencing during SMC phenotypic switching *in vivo*. Circ Res. 2012; 111:685–96. 10.1161/CIRCRESAHA.112.269811 22811558 PMC3517884

[6] Sangwung P, Zhou G, Nayak L, Chan ER, Kumar S, Kang DW, Zhang R, Liao X, Lu Y, Sugi K, Fujioka H, Shi H, Lapping SD, et al. KLF2 and KLF4 control endothelial identity and vascular integrity. JCI Insight. 2017; 2:e91700. 10.1172/jci.insight.91700 28239661 PMC5313061

[7] Zhang X, Wang L, Han Z, Dong J, Pang D, Fu Y, Li L. KLF4 alleviates cerebral vascular injury by ameliorating vascular endothelial inflammation and regulating tight junction protein expression following ischemic stroke. J Neuroinflammation. 2020; 17:107. 10.1186/s12974-020-01780-x 32264912 PMC7140364

[8] Yang Z, Tang X, McMullen TPW, Brindley DN, Hemmings DG. PDGFRα Enhanced Infection of Breast Cancer Cells with Human Cytomegalovirus but Infection of Fibroblasts Increased Prometastatic Inflammation Involving Lysophosphatidate Signaling. Int J Mol Sci. 2021; 22:9817. 10.3390/ijms22189817 34575976 PMC8471290

[9] Lawler PR, Kotrri G, Koh M, Goodman SG, Farkouh ME, Lee DS, Austin PC, Udell JA, Ko DT. Real-world risk of cardiovascular outcomes associated with hypertriglyceridaemia among individuals with atherosclerotic cardiovascular disease and potential eligibility for emerging therapies. Eur Heart J. 2020; 41:86–94. 10.1093/eurheartj/ehz767 31733058

[10] Rea IM, Gibson DS, McGilligan V, McNerlan SE, Alexander HD, Ross OA. Age and Age-Related Diseases: Role of Inflammation Triggers and Cytokines. Front Immunol. 2018; 9:586. 10.3389/fimmu.2018.00586 29686666 PMC5900450

[11] Chi C, Li DJ, Jiang YJ, Tong J, Fu H, Wu YH, Shen FM. Vascular smooth muscle cell senescence and age-related diseases: State of the art. Biochim Biophys Acta Mol Basis Dis. 2019; 1865:1810–21. 10.1016/j.bbadis.2018.08.015 31109451

[12] Beck J, Horikawa I, Harris C. Cellular Senescence: Mechanisms, Morphology, and Mouse Models. Vet Pathol. 2020; 57:747–57. 10.1177/0300985820943841 32744147

[13] Ramírez R, Ceprian N, Figuer A, Valera G, Bodega G, Alique M, Carracedo J. Endothelial Senescence and the Chronic Vascular Diseases: Challenges and Therapeutic Opportunities in Atherosclerosis. J Pers Med. 2022; 12:215. 10.3390/jpm12020215 35207703 PMC8874678

[14] Niu N, Xu S, Xu Y, Little PJ, Jin ZG. Targeting Mechanosensitive Transcription Factors in Atherosclerosis. Trends Pharmacol Sci. 2019; 40:253–66. 10.1016/j.tips.2019.02.004 30826122 PMC6433497

[15] Rowland BD, Peeper DS. KLF4, p21 and context-dependent opposing forces in cancer. Nat Rev Cancer. 2006; 6:11–23. 10.1038/nrc1780 16372018

[16] Xu Q, Liu M, Zhang J, Xue L, Zhang G, Hu C, Wang Z, He S, Chen L, Ma K, Liu X, Zhao Y, Lv N, et al. Overexpression of KLF4 promotes cell senescence through microRNA-203-survivin-p21 pathway. Oncotarget. 2016; 7:60290–302. 10.18632/oncotarget.11200 27531889 PMC5312384

[17] Cheng J, Lin M, Chu M, Gong L, Bi Y, Zhao Y. Emerging role of FBXO22 in carcinogenesis. Cell Death Discov. 2020; 6:66. 10.1038/s41420-020-00303-0 32793396 PMC7385156

[18] Wang G, Han B, Zhang R, Liu Q, Wang X, Huang X, Liu D, Qiao W, Yang M, Luo X, Hou J, Yu B. C1q/TNF-Related Protein 9 Attenuates Atherosclerosis by Inhibiting Hyperglycemia-Induced Endothelial Cell Senescence Through the AMPKα/KLF4 Signaling Pathway. Front Pharmacol. 2021; 12:758792. 10.3389/fphar.2021.758792 34744738 PMC8569937

[19] Huang Y, Zhang H, Wang L, Tang C, Qin X, Wu X, Pan M, Tang Y, Yang Z, Babarinde IA, Lin R, Ji G, Lai Y, et al. JMJD3 acts in tandem with KLF4 to facilitate reprogramming to pluripotency. Nat Commun. 2020; 11:5061. 10.1038/s41467-020-18900-z 33033262 PMC7545202

[20] Jiang F, Chen Q, Wang W, Ling Y, Yan Y, Xia P. Hepatocyte-derived extracellular vesicles promote endothelial inflammation and atherogenesis via microRNA-1. J Hepatol. 2020; 72:156–66. 10.1016/j.jhep.2019.09.014 31568800

[21] Hatzmann FM, Ejaz A, Wiegers GJ, Mandl M, Brucker C, Lechner S, Rauchenwald T, Zwierzina M, Baumgarten S, Wagner S, Mattesich M, Waldegger P, Pierer G, Zwerschke W. Quiescence, Stemness and Adipogenic Differentiation Capacity in Human DLK1-/CD34+/CD24+ Adipose Stem/Progenitor Cells. Cells. 2021; 10:214. 10.3390/cells10020214 33498986 PMC7912596

[22] Sun L, Lin P, Chen Y, Yu H, Ren S, Wang J, Zhao L, Du G. miR-182-3p/Myadm contribute to pulmonary artery hypertension vascular remodeling via a KLF4/p21-dependent mechanism. Theranostics. 2020; 10:5581–99. 10.7150/thno.44687 32373233 PMC7196306

[23] Tong J, Ji B, Gao YH, Lin H, Ping F, Chen F, Liu XB. Sirt6 regulates autophagy in AGE-treated endothelial cells via KLF4. Nutr Metab Cardiovasc Dis. 2022; 32:755–64. 10.1016/j.numecd.2021.12.020 35123854

[24] Rajendran P, Alzahrani AM, Hanieh HN, Kumar SA, Ben Ammar R, Rengarajan T, Alhoot MA. Autophagy and senescence: A new insight in selected human diseases. J Cell Physiol. 2019; 234:21485–92. 10.1002/jcp.28895 31144309

[25] Hsieh MH, Chen YT, Chen YT, Lee YH, Lu J, Chien CL, Chen HF, Ho HN, Yu CJ, Wang ZQ, Teng SC. PARP1 controls KLF4-mediated telomerase expression in stem cells and cancer cells. Nucleic Acids Res. 2017; 45:10492–503. 10.1093/nar/gkx683 28985359 PMC5737510

[26] Ye W, Ni Z, Yicheng S, Pan H, Huang Y, Xiong Y, Liu T. Anisomycin inhibits angiogenesis in ovarian cancer by attenuating the molecular sponge effect of the lncRNA-Meg3/miR-421/PDGFRA axis. Int J Oncol. 2019; 55:1296–312. 10.3892/ijo.2019.4887 31638182 PMC6831202

[27] Ahmed I, Sozmen M. Expression of PDGF-A, PDGFRA, integrin subunit alpha V and selectin E is increased in canine cutaneous fibrosarcomas. Biotech Histochem. 2021; 96:546–54. 10.1080/10520295.2020.1832256 33034211

[28] Chen H, Gu X, Liu Y, Wang J, Wirt SE, Bottino R, Schorle H, Sage J, Kim SK. PDGF signalling controls age-dependent proliferation in pancreatic β-cells. Nature. 2011; 478:349–55. 10.1038/nature10502 21993628 PMC3503246

[29] Sun T, Yin L, Kuang H. miR-181a/b-5p regulates human umbilical vein endothelial cell angiogenesis by targeting PDGFRA. Cell Biochem Funct. 2020; 38:222–30. 10.1002/cbf.3472 31879991

[30] Naipauer J, Rosario S, Gupta S, Premer C, Méndez-Solís O, Schlesinger M, Ponzinibbio V, Jain V, Gay L, Renne R, Chan HL, Morey L, Salyakina D, et al. PDGFRA defines the mesenchymal stem cell Kaposi’s sarcoma progenitors by enabling KSHV oncogenesis in an angiogenic environment. PLoS Pathog. 2019; 15:e1008221. 10.1371/journal.ppat.1008221 31881074 PMC6980685

[31] Ki DH, He S, Rodig S, Look AT. Overexpression of PDGFRA cooperates with loss of NF1 and p53 to accelerate the molecular pathogenesis of malignant peripheral nerve sheath tumors. Oncogene. 2017; 36:1058–68. 10.1038/onc.2016.269 27477693 PMC5332555

[32] Farbehi N, Patrick R, Dorison A, Xaymardan M, Janbandhu V, Wystub-Lis K, Ho JW, Nordon RE, Harvey RP. Single-cell expression profiling reveals dynamic flux of cardiac stromal, vascular and immune cells in health and injury. Elife. 2019; 8:e43882. 10.7554/eLife.43882 30912746 PMC6459677

[33] Yang X, Pande S, Koza RA, Friesel R. Sprouty1 regulates gonadal white adipose tissue growth through a PDGFRα/β-Akt pathway. Adipocyte. 2021; 10:574–86. 10.1080/21623945.2021.1987634 34714716 PMC8565826

[34] Heldin CH. Targeting the PDGF signaling pathway in tumor treatment. Cell Commun Signal. 2013; 11:97. 10.1186/1478-811X-11-97 24359404 PMC3878225

[35] He Z, Chen L, Chen G, Smaldini P, Bongers G, Catalan-Dibene J, Furtado GC, Lira SA. Interleukin 1 beta and Matrix Metallopeptidase 3 Contribute to Development of Epidermal Growth Factor Receptor-Dependent Serrated Polyps in Mouse Cecum. Gastroenterology. 2019; 157:1572–83.e8. 10.1053/j.gastro.2019.08.025 31470007 PMC7006742

[36] Liu L, Wu L, Shan D, Han B. Characterization and clinical relevance of PDGFRA pathway copy number variation gains across human cancers. Mol Genet Genomics. 2022; 297:561–71. 10.1007/s00438-022-01860-y 35212838 PMC8960564

[37] Slattery ML, Herrick JS, Mullany LE, Samowitz WS, Sevens JR, Sakoda L, Wolff RK. The co-regulatory networks of tumor suppressor genes, oncogenes, and miRNAs in colorectal cancer. Genes Chromosomes Cancer. 2017; 56:769–87. 10.1002/gcc.22481 28675510 PMC5597468

[38] Salazar G. NADPH Oxidases and Mitochondria in Vascular Senescence. Int J Mol Sci. 2018; 19:1327. 10.3390/ijms19051327 29710840 PMC5983750

[39] Imai S. Nicotinamide phosphoribosyltransferase (Nampt): a link between NAD biology, metabolism, and diseases. Curr Pharm Des. 2009; 15:20–8. 10.2174/138161209787185814 19149599 PMC2734389

[40] Ma C, Pi C, Yang Y, Lin L, Shi Y, Li Y, Li Y, He X. Nampt Expression Decreases Age-Related Senescence in Rat Bone Marrow Mesenchymal Stem Cells by Targeting Sirt1. PLoS One. 2017; 12:e0170930. 10.1371/journal.pone.0170930 28125705 PMC5268649

[41] Jadeja RN, Powell FL, Jones MA, Fuller J, Joseph E, Thounaojam MC, Bartoli M, Martin PM. Loss of NAMPT in aging retinal pigment epithelium reduces NAD+ availability and promotes cellular senescence. Aging (Albany NY). 2018; 10:1306–23. 10.18632/aging.101469 29905535 PMC6046249

[42] Rosencrans WM, Walsh ZH, Houerbi N, Blum A, Belew M, Liu C, Chernak B, Brauer PR, Trazo A, Olson A, Hagos E. Cells deficient for Krüppel-like factor 4 exhibit mitochondrial dysfunction and impaired mitophagy. Eur J Cell Biol. 2020; 99:151061. 10.1016/j.ejcb.2019.151061 31839365

[43] Skamagki M, Correia C, Yeung P, Baslan T, Beck S, Zhang C, Ross CA, Dang L, Liu Z, Giunta S, Chang TP, Wang J, Ananthanarayanan A, et al. ZSCAN10 expression corrects the genomic instability of iPSCs from aged donors. Nat Cell Biol. 2017; 19:1037–48. 10.1038/ncb3598 28846095 PMC5843481

[44] Sun X, Sun BL, Babicheva A, Vanderpool R, Oita RC, Casanova N, Tang H, Gupta A, Lynn H, Gupta G, Rischard F, Sammani S, Kempf CL, et al. Direct Extracellular NAMPT Involvement in Pulmonary Hypertension and Vascular Remodeling. Transcriptional Regulation by SOX and HIF-2α. Am J Respir Cell Mol Biol. 2020; 63:92–103. 10.1165/rcmb.2019-0164OC 32142369 PMC7328254

[45] Zhu S, Xing C, Zhang G, Peng H, Wang Z. Icaritin induces cellular senescence by accumulating the ROS production and regulation of the Jak2/Stat3/p21 pathway in imatinib-resistant, chronic myeloid leukemia cells. Am J Transl Res. 2021; 13:8860–72. 34540000 PMC8430097

